# Supplementary figures and images for: A meta-analysis of effects of dietary seaweed on beef and dairy cattle performance and methane yield
Source: PLoS One. 2021 Jul 12;16(7):e0249053. doi: 10.1371/journal.pone.0249053 (PMC8274914; doi:10.1371/journal.pone.0249053)

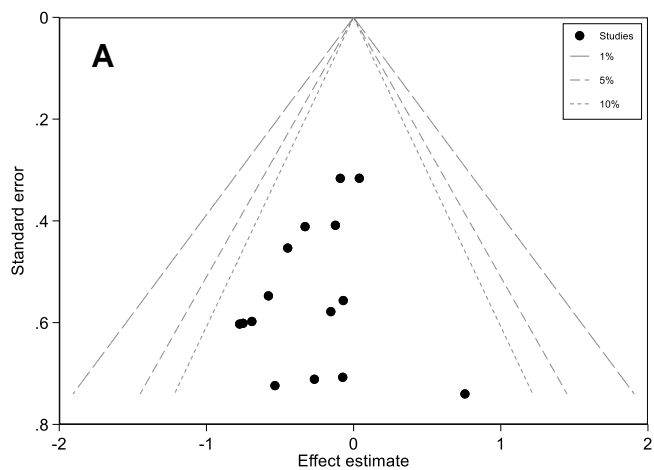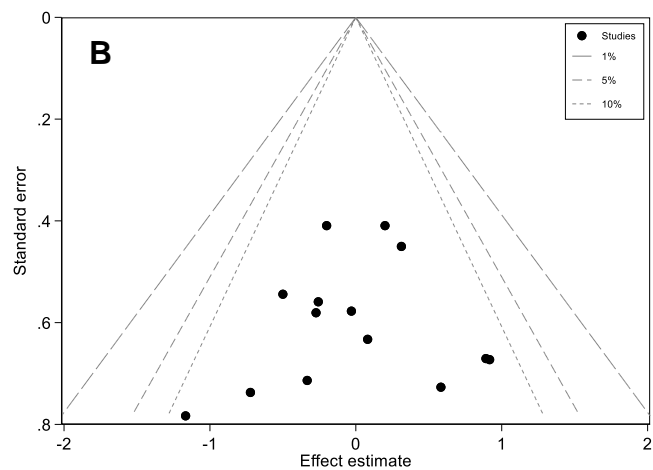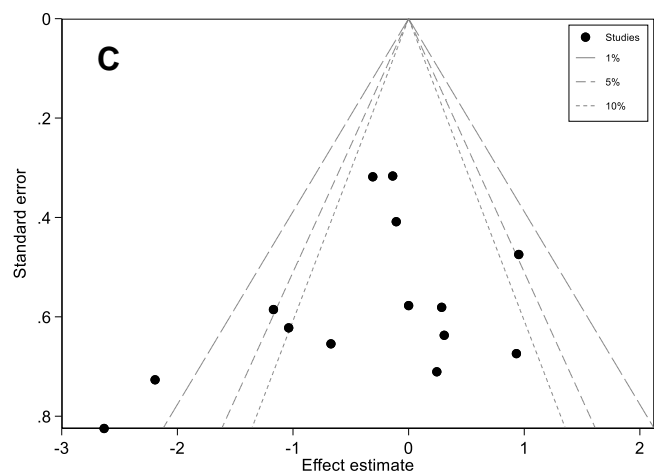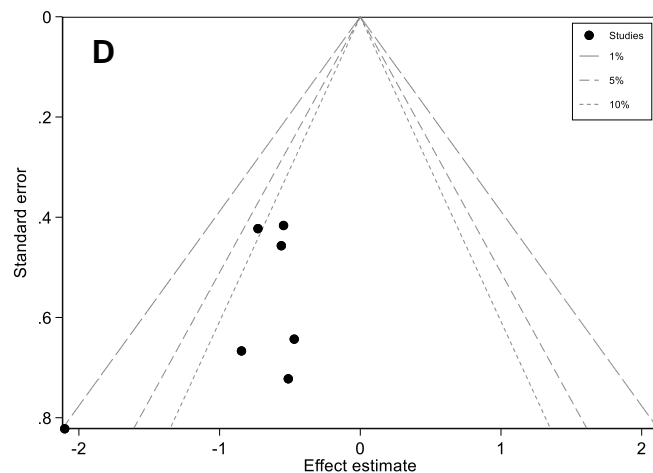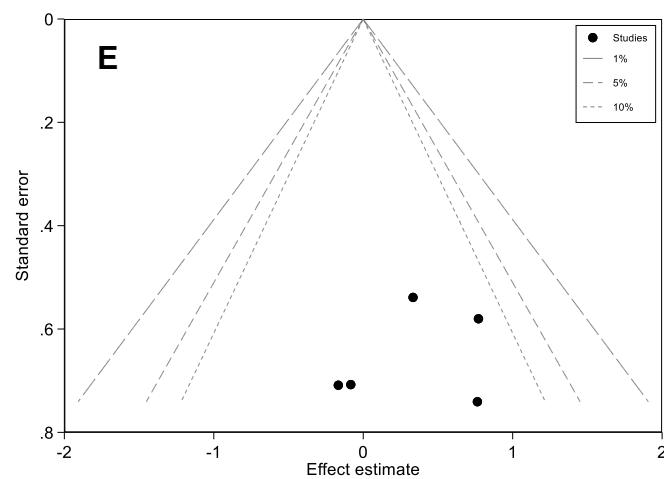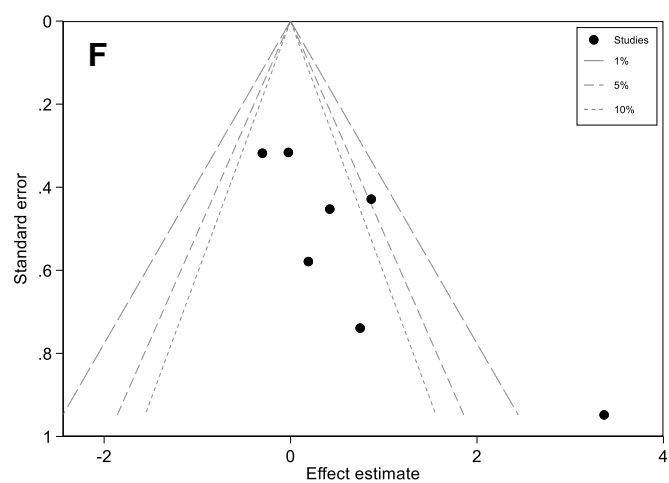

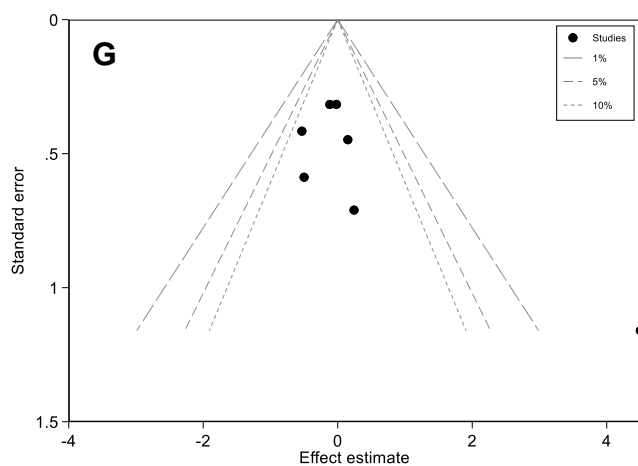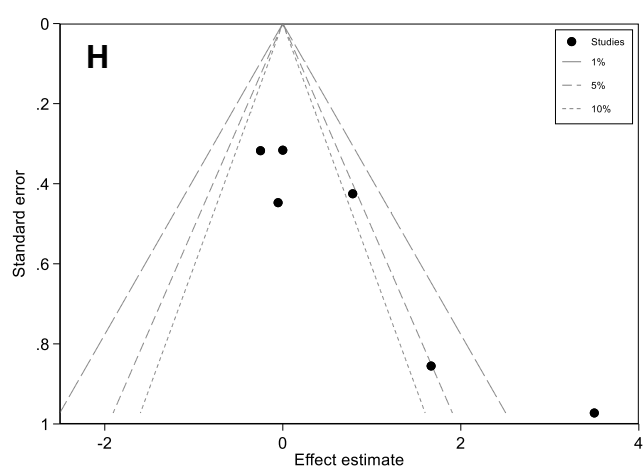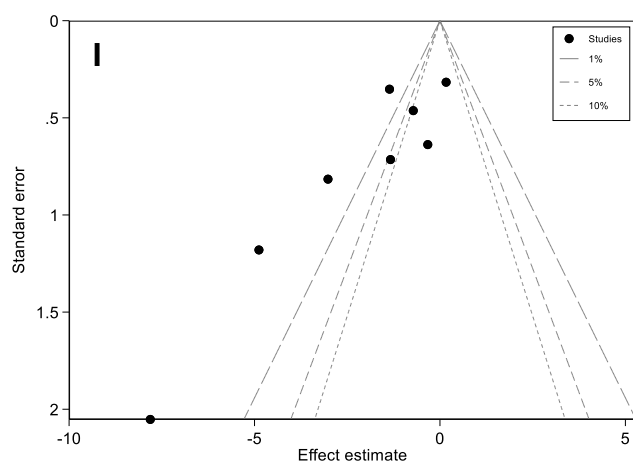

Supplement: S1 Fig — Contour-enhanced funnel plot for effects of seaweed intervention in cattle on (A) final body weight, (B) average daily gain, (C) dry matter intake, (D) feed to gain, (E) gain to feed, (F) milk volume, (G) milk fat percent, (H) milk protein percent, and (I) methane yield. The grey broken lines represent the 90, 95, and 99% CI for treatment comparisons. Effect estimates from small comparisons will scatter more widely at the bottom of the graph and the spread narrows for larger comparisons. In the absence of heterogeneity or bias the plot should approximately resemble a symmetrical (inverted) funnel with comparisons lying within these lines. If there is bias, for example because smaller comparisons without statistically significant effects remain unpublished, this will lead to an asymmetrical appearance of the funnel plot and a gap will be evident in the bottom left-hand corner of the graph. (PDF) [file pone.0249053.s001.pdf]
